# Peripheral neurotrophin levels during controlled crack/cocaine abstinence: a systematic review and meta-analysis

**DOI:** 10.1038/s41598-024-51901-2

**Published:** 2024-01-16

**Authors:** E. Morelos-Santana, D. Islas-Preciado, R. Alcalá-Lozano, J. González-Olvera, E. Estrada-Camarena

**Affiliations:** 1 Laboratorio de Neuromodulación, Subdirección de Investigaciones Clínicas, Instituto Nacional de Psiquiatría Ramón de la Fuente Muñiz, Mexico City, Mexico; 2 Laboratorio de Neuropsicofarmacología, Dirección de Investigación en Neurociencias, Instituto Nacional de Psiquiatría Ramón de la Fuente Muñiz, 101. Col. San Lorenzo Huipulco, CP 14370 Mexico City, Mexico; 3 Secretariado Técnico del Consejo Nacional de Salud Mental, Mexico City, Mexico

**Keywords:** Addiction, Neurotrophic factors

## Abstract

Cocaine/crack abstinence periods have higher risk of relapse. Abstinence as initial part of the recovery process is affected by learning and memory changes that could preserve the addictive cycle. To further understand how the interruption of cocaine/crack consumption affects neurotrophin level we performed the present systematic review and meta-analysis following the PRISMA statement (number CRD42019121643). The search formula was conducted in PubMed, Web of Science, Embase, ScienceDirect, and Google Scholar databases. The inclusion criterion was cocaine use disorder in 18 to 60-year-old people, measuring at least one neurotrophin in blood before and after a controlled abstinence period. Studies without pre-post design were excluded. Five investigations had nine different reports, four of them were subjected to a meta-analysis (n = 146). GRADE risk of bias method was followed. Individual studies reported increased peripheral brain derived neurotrophic factor (BDNF) after abstinence, evidence pooled by Hedge’s *g* showed no significant change in BDNF after abstinence. Relevant heterogeneity in the length of the abstinence period (12–32 days), last cocaine/crack consumption monitoring and blood processing were detected that could help to explain non-significant results. Further improved methods are suggested, and a potential BDNF augmentation hypothesis is proposed that, if true, would help to understand initial abstinence as a re-adaptation period influenced by neurotrophins such as the BDNF.

## Introduction

Cocaine and crack consumption represents a severe public health problem with a 0.42% worldwide prevalence in the general population in 2021, corresponding roughly to 22 million people^1^. Repeated cocaine/crack consumption could elicit a cocaine use disorder (CUD) characterized mainly by a compulsive consumption despite adverse consequences and the presence of craving and withdrawal syndrome, according to the DSM 5^2^. In the USA, 1.483 million people older than 12 years experienced a cocaine use disorder (CUD) in 2021^3^. Daily cocaine consumption, unstable life conditions, and poor social support are associated with elevated mortality risks in the CUD population^4^. No specific approved pharmacological treatment for CUD is available and psychosocial interventions have shown limited efficacy in promoting abstinence^5^. Cocaine/crack abstinence periods are characterized by a high risk of relapse^6^. Because of this, it is necessary to explore neurobiological underpinnings during abstinence stages for a better understanding of the CUD pathogenesis and potential therapeutic interventions.

It is proposed that the establishment of CUD is due to learning and memory related neuroadaptations occurring in the mesocortical-limbic system. These neuroadaptations provoke hypersensitivity to drug-related cues, impulsive decisions making, and other non-adaptive behaviors^7^. Neurotrophins are a group of proteins that modulate neuroadaptation and remodeling processes like cellular surviving, dendritic growth, and synaptic function modifications^8^. This family of proteins includes the nerve growth factor (NGF), brain derived neurotrophic factor (BDNF), neurotrophin 3 (NT3), and neurotrophin 4/5 (NT4/5)^9^.

Other neuroadaptations have been described in long periods without cocaine consumption that could generate a diminution in the seeking behavior^10^. However, short periods without consumption also generate rapid neuroadaptations^11^ and it has been suggested that neurotrophins mediate some of these changes. Increasing evidence about the brain derived neurotrophic factor (BDNF), the most studied neurotrophin in animal models of CUD^12,13^ suggest that this protein participates modulating the reward system structures, such as nucleus accumbens, where it potentiates seeking behaviors whereas its diminution decreases seeking behaviors^10^. After long periods without cocaine consumption, it is suggested that neurotrophins in the reward system participates in reinstalling the seeking behavior in rodents^13^. However, it is unknown whether similar abstinence periods influence neurotrophin levels in humans. Therefore, to understand the neurotrophins levels modifications during cocaine abstinence, we performed a systematic review and meta-analysis to describe how cocaine or crack abstinence periods modify neurotrophin levels in individuals with CUD.

## Methods

This review followed the Preferred reporting items for systematic reviews and meta-analysis and protocols (PRISMA-P) principles^14^ with registration number CRD42019121643.

### Eligibility criteria

Inclusion criteria comprised: (1) Studies including cocaine users or subjects diagnosed with CUD according to a standardized diagnostic criterion, (2) reports including adults, both men and women, in the 18 to 60 years range, (3) studies with a pre-post design with two or more blood samplings, (4) at least one neurotrophin (NGF, BDNF, NT3, NT4/5) measured in any blood component because, blood levels some neurotrophins, such as BDNF, correlate positively with central levels^15^. The intervention of interest was considered an inpatient period in a controlled environment such as hospitalization or ambulatory settings with repeated testing to confirm cocaine abstinence, i.e., urine testing. Exclusion criteria were: (1) Studies without pre-post design, (2) studies with no-controlled abstinence periods, 3) subjects not in the 18 to 60 years range, because BDNF levels may decrease with ageing^16,17^, (4) pre-clinical studies in animal models, (5) studies identified as a systematic review and/or meta-analysis, (6) neurotrophins were sampled in any other tissue or organ other than blood.

### Search strategy

A systematic search formula was designed based on MeSH terms. It included four main components: (a) neurotrophins, (b) cocaine use disorder, (c) abstinence or withdrawal syndrome and (d) exclusion of any substance different from cocaine substances (Supplementary material). This formula was applied and properly adjusted for each of the following databases: Pubmed, Embase, Web of Science, ScienceDirect, and Google Scholar in September 2022 and repeated in March 2023. The searches had no date or language restrictions. For all searches, a .ris file was generated from the database site and for Google Scholar from the software Publish or Perish^18^ ;then, .ris files were processed in the Zotero reference manager (Zotero ver 6.0.23).

### Study selection

**Figure 1:**
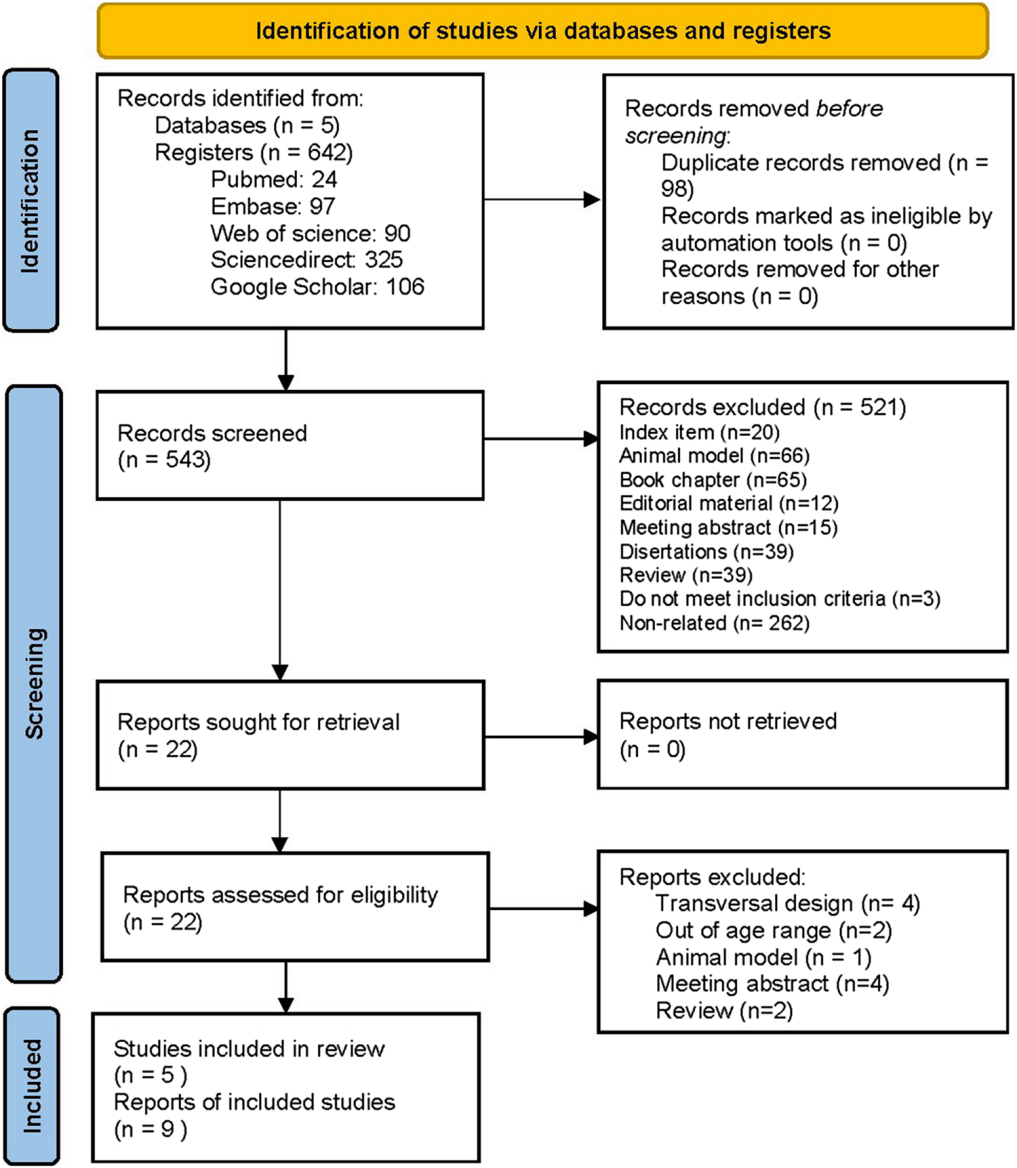
Flow diagram of registers identified in systematic search per stage.

Repeated records detection, title and abstract screenings were performed in Excel. The title and abstract screenings were performed in duplicate independently by DIP and EMS. In the title screening, an item could be excluded if it did not meet an inclusion criterion, or an exclusion criterion was observed. After that, the full-text screening was completed in duplicate by DIP and EMS. If there was a selection inconsistency, JGO, RAL, or EEC resolved it as a third judge. In this step, inclusion criteria were corroborated. In a first revision, a total of 642 records were obtained from the databases examined and duplicated articles were removed (n = 98). A total of 543 titles were screened to detect whether they fulfilled inclusion or exclusion considerations to the next revision round. A total of 521 reports were eliminated due to exclusion parameters detailed in the flow chart (Fig. 1). Once excluded, a total of 22 reports were sought for retrieval for full text examination by two reviewers (EMS and DIP, Kappa index 0.83). After a complete revision of the reports, a total of 13 articles were excluded due to reasons detailed in the flow chart (Fig. 1). Finally, nine reports were included in the present systematic review, and four of them were subjected to a meta-analysis.

### Data extraction

An evidence table was constructed summarizing the main characteristics of the included studies (i.e. objectives, sample included, outcomes regarding neurotrophin levels, conclusions). To reduce potential bias or omissions, the information was condensed independently in pairs by EMS and DIP. At this point, we detected that nine reports belonged to five different investigations and the data from the nine studies were fully extracted. Only one report per investigation was considered for the meta-analysis to avoid the inclusion of repeated participants. Four reports were submitted to a meta-analysis and a fifth report was excluded because mean values were missing, and it was impossible to obtain the data despite efforts to contact the authors. The four articles subjected to the meta-analysis had a pre-post design. A standardized mean difference was used as a statistical approach to compare each report. We extracted raw means and standard deviations from every article to compare neurotrophin levels at pre and post inpatient period. Once raw values were extracted, a standardized mean difference (Cohen’s d) and a correction factor (*Hedge’s j*) was calculated^19^. Finally, to reduce potential overestimations in effect sizes, we are reporting Hedge’s g, as it is considered an unbiased estimator of mean differences^19^. All data extraction and calculations were performed in Excel.

The meta-analysis was performed using Review Manager (RevMan) software, ver. 5.3^20^. Data were analyzed through the generic inverse variance with the standard mean difference method. All analyses were performed by random effects based on Higgins’ I^2^ heterogeneity coefficient (> 50% in all cases). P values < 0.05 were considered as significant.

## Quality of evidence

### Method

**Figure 2:**
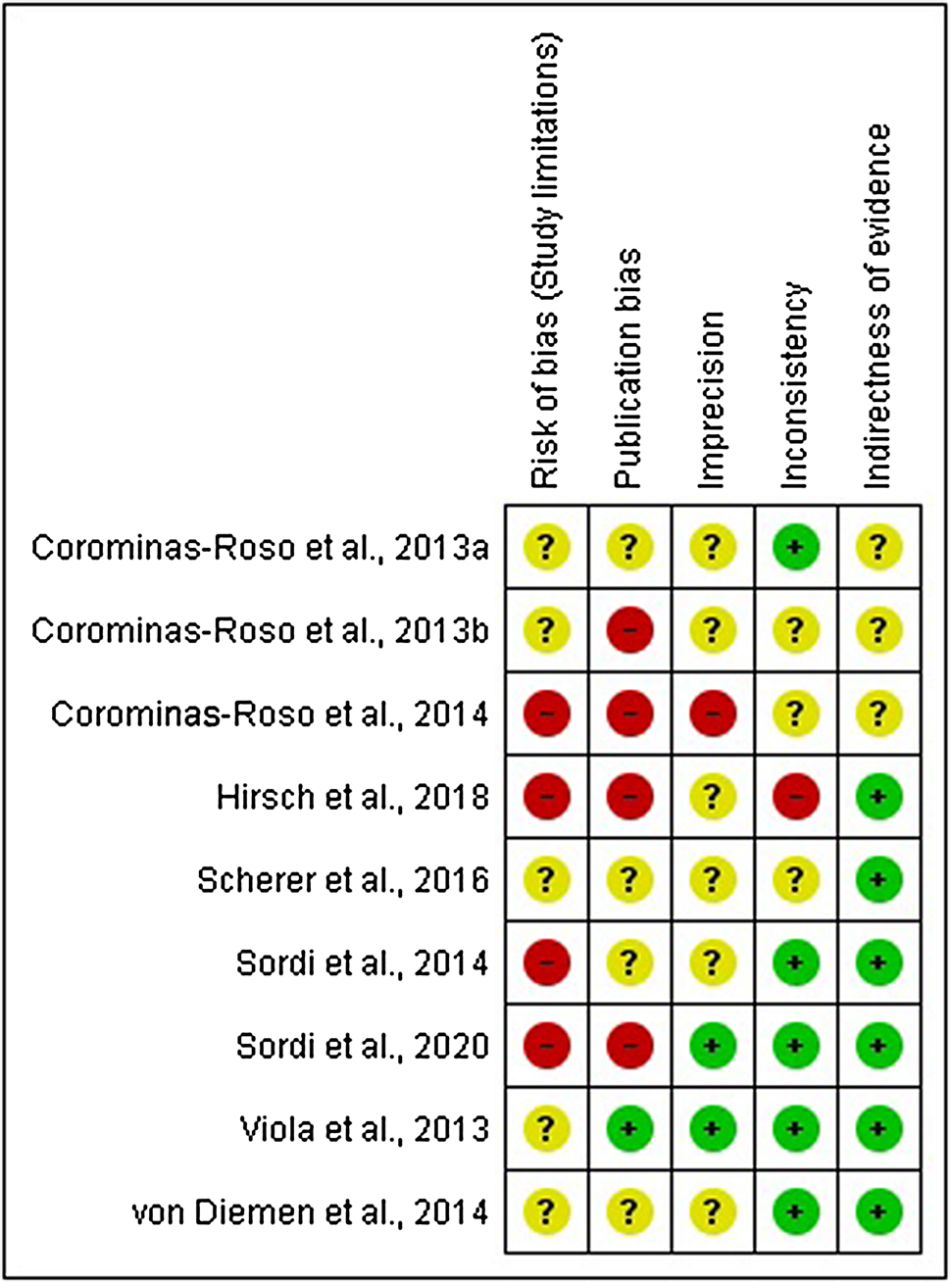
Grading of Recommendations, Assessment, Development and Evaluation (GRADE) system criteria for quality of evidence assessment of the 9 included reports.

The Grading of Recommendations, Assessment, Development and Evaluation (GRADE) system criteria were followed to rate the quality of evidence included in the present work^21–26^. We considered the following criteria: (1) risk of bias regarding study limitations, (2) publication bias (3) imprecision, (4) inconsistency of results and (5) indirectness of evidence. The five previous criteria were rated as low risk, unclear risk, or high risk. The bias results were personalized using the RevMan 5.3 software, and results are summarized in Fig. 2.

#### Risk of bias (study limitations)

All articles included were observational pre-post design studies. The reports by Corominas-Roso et al.^27^, Hirsch et al.^28^ and two reports by Sordi et al.^29,30^ were rated as high risk of bias, as several limitations were detected (mainly wide inclusion/exclusion criteria, low sample, not confirming CUD diagnosis). Corominas-Roso et al.^31,32^, Viola et al.^33^, Scherer et al.^34^ and von Diemen et al.^35^ reports were rated as unclear risk as they mitigated the influence of potential confounders, and most participants were diagnosed with CUD and confirmed cocaine consumption before the inpatient period through a positive urine test. Most of the studies included only men or an unrepresentative number of women. Therefore, the results should be interpreted with this limitation in mind.

#### Publications bias

Viola et al.^33^ report was rated as low risk because clear objectives were stated, statistical analysis included covariables control that was in line with the aim of the study. No selective or other bias was detected. Corominas-Roso et al.^32^ was rated as unclear risk because a non-parametric test was used based on low sample instead of a normality test. Posterior Corominas-Roso et al.^27,31^ reports were rated as high risk as we detected the same recruitment period and trial registration, but this was not disclosed in the articles. Similarly, authors did not disclose the same recruitment period in Sordi et al.^29,30^ and von Diemen et al.^35^; however, additional outcomes were addressed, and statistical methods were appropriately conducted. Thus, Sordi^30^ and von Diemen et al.^35^ reports were rated as unclear risk. Besides the former risk, Sordi et al.^29^ was rated as high risk as the discussion focused on a low correlation value that apparently is not the central interest when the study is introduced. Scherer et al.^34^ was rated as unclear risk considering that the discussion is focused on the significant results and seems to overlook non-significant results that were not in line with the hypothesis. Hirsch et al.^28^ was rated as high risk as the authors omitted statistical values that affect the interpretation of their results.

A funnel plot followed by Egger's test were performed to screen potential publication bias in four studies included in the meta-analysis (funnel plot included in Supplementary Material). Egger's test revealed no significant asymmetry (t = − 0.27, df = 2, p = 0.81), suggesting no publication bias in the articles included.

#### Imprecision

A low risk of imprecision was observed in Viola et al.^33^ and Sordi et al.^29^ because they showed a sufficient number of subjects, reasonable statistical control, C.I.s, and effect sizes. The study by Corominas-Roso et al.^27^ was rated as a high risk of imprecision, due to the high variability observed in the relapse days to cocaine consumption in one of the evaluated groups, which could affect the inter-subjects comparability. The remaining studies^28,31,32,34–36^ were rated as unclear risk of imprecision mainly because of the small sample and omission of C.I.s.

#### Inconsistency

The reports from Corominas et al.^32^. Sordi et al.^29,30^, and Viola et al.^33^ and von Diemen et al.^35^ were rated as low of risk of inconsistency due to their findings are aligned showing similar increases in BDNF after inpatient period. Corominas-Roso et al.,^27,31^ and Scherer et al.^34^ were rated as unclear risk because their results partially confirm BDNF increases in some of the included subgroups included i.e., no-psychosis history^31^ or inpatient completer group^34^. Hirsch et al.^28^ was rated as high risk of inconsistency because they reported decreased BDNF after inpatient period, opposite results compared to the rest of studies reviewed.

#### Indirectness of evidence

Low risk of indirectness was observed in the studies by Viola et al.^33^, Sordi et al.^29,30^, von Diemen et al.^35^, Scherer et al.^34^ and Hirsch et al.^28^, as all of them evaluated neurotrophins (BDNF mainly) in cocaine abstinence as main outcome. The Corominas-Roso et al. studies^27,31,32^ were rated as unclear risk or indirectness as data were collected from subjects enrolled in a clinical trial whose main objective was other than to evaluate neurotrophin levels.

In summary, the main concerns might be the study limitations and publication bias. All the studies reviewed started as unclear risk due to the experimental design and the lack of statistical control for confounders. Additionally, omissions about the recruitment periods in two investigations revealed potential publication bias.

### Ethics approval and consent to participate

The authors of the reviewed studies declared approval from local Ethics Committee to perform the study and stated a written informed consent by participants in all cases.

## Results

### Characteristics of included articles

The following articles were finally included in the systematic review: Corominas-Roso et al.^27,31,32^, Viola et al.^33^, Scherer et al.^34^, von Diemen et al.^35^, Hirsch et al.^28^ and Sordi et al.^29,36^. Due to possible sample duplication, only one report per study was included in the meta-analysis. The articles finally subjected to the meta-analysis were: Corominas-Roso et al.^32^, Scherer et al.^34^, von Diemen et al.^35^, and Hirsch et al.^28^.

Six of the studies reviewed were conducted in Brazil^28,29,33–36^, and the rest were performed in Spain^27,31,32^. As mentioned previously, only studies with pre-post design were included in the present work. Cocaine dependence in Corominas-Roso et al.^27,31,32^ and Viola et al.^33^ reports was diagnosed based on DSM-IV criteria^37^ using the Structured Clinical Interview for DSM axis I disorders (SCID-I). Reports from Scherer et al.^34^, von Diemen et al.^35^, Sordi et al.^29,36^ declared the use of SCID-I and Hirsch et al.^28^ the use of the Mini International Neuropsychiatric Interview test (MINI) to inquiry about cocaine consumption and psychiatric symptomatology but no formal CUD was stated in their reports.

The samples were constituted mainly by men, except for Viola et al.^33^ who included only women, and none of the reviewed articles included biological sex as a co-factor in the statistical analysis. All articles included adults from > 18 to < 45 years, most of them with < 8 years of formal education, mostly Caucasians, although Viola et al.^33^ did not detail ethnicity of the participants. All subjects underwent a controlled inpatient period. Demographic results are reported in Table 1.

**Table 1:** Main socio-demographic characteristics and findings of reviewed studies.

Study	N		Demographics			Consumption characteristics				Inpatient period	Sample collection and processing		Neurotrophin levels		Summary of findings
	Total	Groups	Age	Sex	Years of education	Years of consumption	Age of consumption onset	Consumption pattern (days in last month)	Amount of cocaine/crack consumed (last month or week)		Blood collection respect last consumption	Clotting	Pre (ng)	Post (ng)	
Corominas-Roso et al., 2013a	19	N/A	33.65 ± 6.85	M: = 21, W = 2	N/A	N/A	23.95 y.o ± 13.65^a^	20.77 ± 7.55^a^	N/A	12 days	1 day after last consumption	2 h	51.67 ± 17.50^a^	60.64 ± 22.60^a^*	**↑**Significant increase in BDNF
Corominas-Roso et al., 2013b	40	HPS n = 18, no-HPS n = 22	HPS 34.18 ± 8.54 no-HPS 33.65 ± 6.8	HPS M = 17 W = 1 no-HPS M = 20 W = 2	N/A	HPS ~ 11^b^ no-HPS ~ 10	HPS 23.29 ± 8.27^a^ no-HPS 24.05 ± 13.99^a^	HPS 18.72 ± 8.97^a^ no- HPS 21.14 ± 7.55^a^	N/A	12 days	1 day after last consumption	2 h	HPS 55.64 ± 25.38^a^ no- HPS 50.19 ± 16.37^a^	HPS 50.70 ± 22.40^a^ no-HPS 58.91 ± 21.92^a^	**↑**Only in no-HPS
Corominas-Roso et al., 2015	40	ER to cocaine consumption n = 20, LR to cocaine consumption n = 20	ER 32.5 (30–38)^c^, LR 32 (28.3–36.5)^c^	M = 38, W = 2	N/A	ER ~ 6.5^b^, LR ~ 5.5^b^	ER 25.5 (21–30.3)^c^, LR 27 (18.8–32.8)^c^	ER 25 (20–30)^c^, LR 20 (10–21)^c^	N/A	12 days	1 day after last consumption	2 h	ER 56.9 (47.4–67.1)^c^, LR 47.1 (32.9–57.7)^c^	ER 49 (44.4–74)^c^, LR 55.1 (33.1–67.4)^c^	**↑**Only in LR
Viola et al., 2014	104	CSA + n = 22, CSA- n = 82	CSA + 31.8 ± 6.2, CSA- 28.1 ± 7.6^a^	W = 104	N/A	CSA + ~ 12, CSA- ~ 10^b^	CSA + 19.2 ± 6.4, CSA- 18.5 ± 5.2^a^	CSA + 15.6 ± 13.5, CSA- 16.2 ± 13.2^a^	NA	21 days	~ 7 days after last consumption	0.5 h	NA	NA	**↑**−GDNF and NT-4/5 increase over time only in CSA + **=**NGF, NT-3 and BDNF no change overtime
Von Diemen et al., 2014	49	NA	27.9 ± 7.38^a^	M = 49	65.3% ≤ 8 years	5 (3–7) ^c^	~ 22 ^b^	N/A	120 (28.6–285.7)^c^	18.3 ± 4.1^a^	Between 1–3 days after last consumption^b^	Immediately	28.6 ± 11^a^	35.5 ± 12.3^a^	**↑**Significant increase in BDNF
Sordi et al., 2014	49	Less severe crack consumption n = 25 , More severe crack consumption n = 23	27.9 ± 7.38^a^	M = 49	65.3% ≤ 8 years	5 (3–7) c	~ 22 b	N/A	120 (28.6–285.7)^c^	18.3 ± 4.1^a^	Between 1–3 days after last consumption^b^	Immediately	28.6 ± 11^a^	Less severe: 38.9 ± 12.08 , More severe: 30.45 ± 11.06^a^	**↑**Negative correlation between BDNF and severity of crack use at discharge. Higher levels of BDNF in less severe crack use
Sordi et al., 2020	33	Low CTE n = 11, Medium CTE n = 11, High CTE n = 11	Low CTE 29.64 ± 8.3, Medium CTE 26.09 ± 5.68, High CTE 25.45 ± 6.47^a^	M = 33	N/A	Low CTE 7 ± 3.5, Medium CTE 6.91 ± 3.51, High CTE 5.82 ± 2.64^a^	Low CTE ~ 23, Medium CTE ~ 19, High CTE ~ 21^b^	N/A	N/A	Low CTE 19.82 ± 4.33, Medium CTE 19.18 ± 4.17, High CTE 17.91 ± 4.39^a^	Between 1–3 days after last consumption^b^	Immediately	Low CTE 29.28 ± 11.62, Medium CTE 26.01 ± 13.08, High CTE 30.77 ± 8.13^a^	Low CTE 32.48 ± 9.88, Medium CTE 33.86 ± 13.09, High CTE 42.99 ± 12.13^a^	**↑**Significant increase in BDNF, regardless of levels of trauma experiences
Scherer et al., 2016	47	Completers of inpatient treatment n = 24, No-completers of inpatient treatment n = 23	Completers 33.58 ± 8.63, No-completers 32.48 ± 8.52^a^	M = 47	61.70% ≤ 8 years	Completers 9.58 ± 5.04, No-completrs 7.96 ± 5.02^a^	Completers 22.5 (17–31.5), No-completers 21 (17–30) ^c^	N/A	Completers 310 (185–600), No-completers 220 (120–500) rocks ^c^	Completers 32.3 ± 4.69, No-completers 20.09 ± 8.80^a^	Between 1–4 days after last consumption^b^	Immediately	Completers 16.85 ± 3.24. No-completers 14.65 ± 5.45^a^	Completers 18.10 ± 4.88, No-completers 13.91 ± 4.77^a^	**↑**Significant increase in BDNF levels only in the "completers" group
Hirsch et al., 2018	31	N/A	28 ± 8.74 ^c^	M = 31	51.6% ≤ 9 years^b^	55.6% ≤ 3 years^b^	N/A	N/A	~ 78 rocks per month ^b^	14 days	N/A	Immediately	363.7 ± 98.20^a^	295.5 ± 106.4^a^	**↓**Significant decrease in BDNF levels

*HPS* history of psychotic symptoms, *no-HPS* no history of psychotic symptoms, *ER* early relapsers to cocaine consumption, *LR * later relapsers to cocaine consumption, *CSA+* history of childhood sexual abuse, *CSA−* no history of childhood sexual abuse, *CTE* childhood trauma experience.

^a^Mean ± standard deviations.

^b^Imputated values from the original report.

^c^Median (interquartile range).

Regarding studies that reported statistical control for co-variables, it was found that Sordi et al.^29^ controlled the neurotrophin results for severity of cocaine consumption and inpatient days. In Viola et al.^33^, the main results were controlled for body mass index (BMI), age and severity of depression. In Scherer et al.^34^, results were statistically controlled for lithium intake, as BDNF levels had higher variations in subjects under this medication. For the rest of the included reports^35,28,32,31^ no statistical control for co-variables was reported.

### Consumption status at the beginning of the inpatient period

Studies from Scherer et al.^34^, von Diemen et al.^35^, and Sordi et al.^29,36^ confirmed cocaine consumption through a positive urine test at the beginning of the inpatient period. The reports by Corominas-Roso et al.^27,32,31^ mention that the last cocaine consumption occurred one day before the inpatient period, but no urine or blood test was performed for cocaine screening. Viola et al.^33^ stated that last cocaine consumption occurred ~ 7 days before, whereas in Hirsch et al.^28^ this information was omitted. Neither Viola et al.^33^ nor Hirsch et al.^28^ screened urine or blood to confirm the last cocaine consumption.

### Severity and pattern of crack/cocaine consumption

The severity of cocaine consumption was assessed through the Addiction Severity Index-6th (ASI-6) in the following reports^29,34,28,36,33,35^ and with the Cocaine Selective Severity Assessment (CSSA) for the rest of the studies^27,32,31^. Regarding the form of cocaine consumed (i.e. powder or crack), powder cocaine consumption was reported in Corominas-Roso et al.^27,32,31^, and crack-cocaine was reported in the remainder reports^29,30,34,28,33,35^.

Moreover, differences in the crack/cocaine consumption pattern were observed among the reviewed studies. In Corominas-Roso et al.^27,32,31^, authors reported that sample subjects consumed ~ 20 days in the last month. The studies by von Diemen et al.^35^ and Sordi et al.^29,36^ reported a median of 120 and an interquartile range (IQR) of 28.6–285.7 rocks used in the last month. Scherer et al.^34^ reported a median of 300 and IQR of 150–600 rocks used in the last month. Viola et al.^33^ reported ~ 15 days of cocaine consumption in the last month. Lastly, Hirsch et al.^28^ reported 18 (± 11) rocks used per week. Therefore, a total of 78 rocks are estimated per month, although authors did not specify if this corresponds to the last week or month. Details of cocaine consumption pattern are reported in Table 1.

### Characteristics of the inpatient period

Controlled abstinence throughout inpatient period varied among the reviewed studies. Although all subjects underwent a controlled inpatient period, differences in length of abstinence were observed. In Corominas-Roso et al.^27,32,31^ an inpatient period of 12 days was stated in the protocol. Von Diemen et al.^35^ and Sordi et al.^29,36^ reported around 18 (± 4) days of inpatient period (for further details see Table 1). In Viola et al.^33^, a 3-week inpatient period was stated. In Scherer et al.^34^, the experimental design comprised two sub-groups divided into “completers” and “non-completers” of a detoxification program. Therefore, inpatient period for “completers” was ~ 21 (± 7) days, and for “non-completers” was ~ 31 (± 4) days. Lastly, in Hirsch et al.^28^, the controlled abstinence period corresponded to 14 days of inpatient period.

### Sample collection and procedure

In all studies, blood samples were collected at least twice: at the beginning of inpatient period and at discharge. All reports stated that the first blood sample was collected at the admission day^27,29,31^, except for Viola et al.^33^, who stated that the first blood sample was collected on day 4 of the inpatient period, a second one on day 11, lastly a third collection was obtained on day 18. The reports that stated the collection of post-inpatient blood sample 24 h before discharge were^29,34–36^. The post-inpatient blood sample was collected after 12 days in Corominas-Roso et al.^27,32,31^ and after 14 days in Hirsch et al.^28^ but these authors did not specify the exact day or timing respect to discharge (i.e. the day of discharge or the day before).

Once the blood sample was collected, a clotting period was allowed that varied among the reviewed studies. In Corominas-Rosso et al.^27,32,31^, a two-hour period of clotting was reported. Viola et al.^33^, allowed 30 min for sample clotting. In contrast, for the rest of the studies^29,34–36^ no clotting period was allowed. After clotting and centrifuging blood samples, all the studies reported the extraction of serum^27,29,31^, and only Viola et al.^33^ extracted plasma to measure neurotrophins.

### Pre-post neurotrophins measures

BDNF was the most common neurotrophin evaluated among the studies included in this analysis. However, NGF, NT3, NT4/5 were additionally measured in Viola et al.^33^. Corominas-Rosso et al.^32^ (n = 19) found that early cocaine abstinence increased BDNF, with statistical differences between pre and post measures (means of BDNF are detailed in Table 1). Moreover, in the following report from Corominas-Rosso et al.^31^ (n = 40), authors found a significant BDNF increase in subjects that never experienced psychotic symptoms and no change was observed in patients with psychotic symptoms at least once during cocaine consumption. Similarly, in the third report from Corominas-Rosso et al.^27^ (n = 40) early cocaine abstinence increased BDNF levels, but this was true only for subjects classified as “late relapsers”, and no change was observed in those classified as “early relapsers” after the inpatient period.

The reports by von Diemen et al.^35^ and Sordi et al.^36^ (n = 49) revealed no significant BDNF increase between pre and post inpatient periods. However, lower levels were observed at inpatient when compared to control non-crack users, and BDNF levels were equivalent between users and control subjects at discharge. Specifically, in Sordi et al.^36^ authors established a sample cutoff between “less severe” and “more severe” crack consumption. Interestingly, a negative correlation showed that those subjects with a more severe crack usage had lower BDNF levels at discharge. In a later report^29^ (n = 33), authors found a significant increase in BDNF levels after the inpatient period and classified the subjects based on childhood trauma experiences as “low”, “medium” and “high”, but no differences in BDNF levels were observed due to trauma experiences.

Viola et al.^33^, classified the sample by those subjects who had childhood sexual abuse history, and found that NT3 and NGF were lower in women with crack dependence when compared to control subjects, but remained unchanged over time (between day 4 and 18) regardless of sexual abuse. BDNF was higher in women with crack dependence in comparison to controls, but remained unaltered over time (between day 4 to 18). However, NT4/5 and GDNF levels increased over time only in subjects with a history of childhood sexual abuse, but they were not different between women with crack dependence and controls.

Scherer et al.^34^ split the sample between “completers” and “non-completers” of the inpatient period who had ~ 31 or ~ 20 days of abstinence, respectively. Authors reported a tendency to higher BDNF levels in the “completer” group at inpatient admission. Moreover, BDNF levels significantly increased at discharge only in the “completers”.

Lastly, in contrast to the reports in the previous articles reviewed in the present work, Hirsch et al.^28^ (n = 31) found that means of BDNF levels decreased significantly after 14 days of detoxification. Discrepancies in the results observed among studies will be discussed in a following section.

### Meta-analysis results

**Figure 3:**
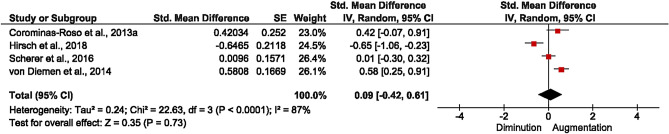
Forest plot of meta-analysis of 4 included studies.

As mentioned previously, four reports (total sample n = 146 participants) were included in the meta-analysis. Only BDNF levels were pooled as no other neurotrophin was measured in more than one study. Figure 3 illustrates the forest plot showing the effect sizes and confidence intervals (C.I. 95%). Data are presented as Hedge’s g and were analyzed by random effects as heterogeneity I2 = 87%. As observed, the analysis’ weight was distributed from lowest, Corominas-Rosso et al.^32^, (23%) to the highest Scherer et al.^34^ (26.4%). As a whole, results showed that controlled cocaine abstinence does not modify BDNF peripheral levels when compared at pre and post-inpatient period (SMD = 0.09, CI [− 0.42, 0.61], p = 0.73).

## Discussion

Nine articles belonging to five different studies were included in the present systematic review. Four reports were subjected to a meta-analysis showing that crack/cocaine abstinence does not significantly modify peripheral BDNF levels between pre- and post-inpatient periods. Regarding quality of evidence, main concerns were identified as risk of bias (study limitations) and publication bias.

We detected relevant heterogeneity regarding the day that the blood sample was taken with respect to the last cocaine or crack consumption among studies. The range of days from the last urine-confirmed consumption goes from 1 day from Corominas-Roso et al.^27,32,31^, 1–3 days reported by von Diemen et al. and Sordi et al.^29,30,35^ and ~ 7 days stated but not urine-confirmed in Viola et al.^33^. In Hirsch et al.^28^, this information was omitted. The time point of sample collection is relevant, as BDNF levels may rapidly change. BDNF levels can vary depending on the time elapsed since cocaine consumption. Animal models have revealed that after 7 days of a single cocaine injection in rats provokes a plasma BDNF protein upregulation^38^. In chronic cocaine consumption, blood collection and cocaine administrations was tested in two different conditions, concurrent and non-concurrent, the plasma BDNF was increased in concurrent condition but decreased in the no-concurrent^39^. Taken together, these findings suggest that the time since the last cocaine exposure may induce heterogeneity in BDNF peripheral levels respect to the last cocaine consumption. Future studies are encouraged to strictly control the time since the last cocaine consumption when assessing BDNF peripheral levels.

Furthermore, different abstinence periods were detected among studies, ranging from 12 days reported in Corominas-Roso et al.^27,32,31^ to 32 days stated in Scherer et al.^34^ respect to the beginning of the inpatient period. The study by Scherer et al.^34^ is the longest BDNF monitoring abstinence period in CUD that we found, they reported that longer cocaine abstinent period induced a BDNF increase in comparison to a shorter period. Longer abstinence periods have been monitored and showed robust changes after 6 to 9 months of alcohol^40^ or heroin^41^ withdrawal. It might be feasible to suggest that a > 32 days of cocaine abstinence period, could increase peripheral BDNF levels. In the present meta-analysis, noticeably shorter and heterogeneous periods of abstinence were analyzed, and this could help to explain the non-significant effects of cocaine abstinence on BDNF levels.

An additional factor is the differences in blood sample processing among reviewed studies regarding clotting time. This heterogeneity may induce additional variability in samples because the coagulation time affects the BDNF levels, increasing detectable levels by augmenting the coagulation time^42–44^. Also, BDNF is better detected in serum than in plasma samples^45^. In this sense, the included studies included reported none^28,34,35^ or 120 min^27,32,31^ coagulation time to obtain serum or 30 min to obtain plasma^33^, which could further explain the non-significant results in our meta-analysis.

Moreover, the included studies considered some co-variables that may influence peripheral BDNF levels. For instance, Sordi et al.^29^ controlled their neurotrophin results for severity of cocaine consumption and inpatient days. In Viola et al.^33^, the main results were controlled for body mass index (BMI), age and severity of depression. As previously reviewed, peripheral BDNF increases as depressive symptoms improve^46^ and BMI has a negative correlation with peripheral BDNF^47^. Scherer et al.^34^ results were statistically controlled for lithium intake, as BDNF levels had higher variations in subjects under this medication, which could be because lithium upregulates BDNF levels^48^. Unfortunately, in an important number of studies included in this review did not report a statistical control for co-variables^27,31^.

On the other hand, severity of cocaine consumption was considered only in some studies. Comparisons between powder and crack cocaine revealed that usually crack consumers have higher addiction severity and higher social dysfunction^49,50^. In the studies reviewed, years of cocaine/crack consumption range is from ~ 3 to12 years and we can consider those participants as chronic cocaine/crack users, BDNF might change differently in more severe consumption. A study non-included in the present review due to a lack of pre-post design, higher peripheral BDNF levels was significantly associated with an early relapse and higher amount of cocaine consumed after 8 weeks of discharge^51^. Additionally, a significant negative correlation between years of consumption and BDNF was reported by Scherer et al.^34^. The present work did not consider samples of healthy controls, but a previous meta-analysis has reported significant lower BDNF levels between cocaine/crack consumers compared to healthy controls^45^. Data suggest that BDNF basal levels could be low and fluctuations along an abstinence period could be associated with severity. Hence, future studies should consider severity as a relevant co-variable when assessing peripheral BDNF in CUD.

Other factors that may have influence in BDNF fluctuations is age, as younger people have higher levels of BDNF and decline with the age, which has been described as a negative correlation in healthy controls in a 20 to50 year old range population^17^. A study not-included in the systematic review due to the age range found a significant increase in BDNF levels in adolescent cocaine-crack consumers after 21 days of abstinence^52^. Then, the study of BDNF in younger cocaine-crack users could help to elucidate how abstinence could change BDNF levels as less confounders like time or severity of consumption modulate the response of BDNF.

Moreover, adverse experiences during childhood, such as physical abuse, increased the risk to develop CUD^53^, however, a previous history of trauma or sexual abuse described in Viola et al.^33^ and Sordi et al.^29^, respectively, showed no effect on BDNF fluctuations between victims/no-victims of those adverse events, but Viola et al.^33^ reported that BDNF was increased in CUD women compared healthy controls. These findings should be expanded by considering the types, chronicity, and number of adverse experiences in childhood to explain possible interactions between adversity and BDNF. For GDNF and NT4/5, Viola et al.^33^ detected an important increase in those patients with a history of childhood sexual abuse. These data highlight the need to consider sex differences and other neurotrophins in response to the abstinence period, as well as stress history as a factor that could contribute to modify the response to abstinence.

Recently it was reported the ratio of proBDNF/BDNF as potential biomarker of CUD^54^. In this report, Miuli et al. controlled comparisons by time-consumption in a sample n = 24. They reported a low proBDNF/BDNF ratio associated with a longer history and higher amount of cocaine use, agreeing with other reports^45^. A lower ratio may be indicative of a BDNF reduced metabolism, possibly related to enzymes and genes modified by cocaine^55^, and they suggest the proBDNF/BDNF ratio as a reliable biomarker for CUD as has been proposed for other psychiatric disorders^56^.

Polymorphisms have been identified differences in the activity of BDNF in animal models of CUD. For example, homozygous VAL/VAL and heterozygous VAL/MET have shown different extinction responses. Heterozygous animals show an improved cocaine-related learning extinction^57^. Other studies reported that repeated cocaine self-administration increases BDNF protein in the reward system by the hyperacetylation of the *Bdnf* promoter (extensively reviewed^58^). Preclinical reports found that BRD4, a facilitator of gene transcription, was increased in the NAc following repeated cocaine exposure^59^. Inhibiting BRD4 reduces the rewarding effect of cocaine^59^ at the time decreasing BDNF protein and mRNA expression^60^.

Moreover, a transgenerational epigenetic increment in acetylation of the *Bdnf* transcriptor and BDNF protein levels in cocaine-sires male offspring^61^^.^. The elevation of BDNF levels was found in mPFC and was related to cocaine-seeking reduction in cocaine-sires male offspring but not in females^61^, showing a sex-dependent epigenetic modulation of the BDNF expression regarding cocaine consumption. The studies reviewed, that measured BDNF during abstinence did not analyze some polymorphisms or epigenetic markers, which could help to describe different profiles of response to abstinence in CUD.

Animal models of cocaine chronic consumption have shown gradual increases in BDNF in the ventral tegmental area (VTA) after 7 days^62^ and 10 to 15 days^63^, in the hippocampus at 9 days^64^, in the nucleus accumbens, and amygdala after 30^65^, and in the medial prefrontal cortex (mPFC) at 90 days of abstinence^66^. The BDNF increases in the reward system have been consistently related to incubation of craving^65,67–69^ and reinstatement of cocaine seeking behavior^70^. This evidence supports that BDNF elevations in the reward system could favor the long-lasting changes that preserve the addictive cycle over time^60^. Therefore, it is feasible to suggest a higher increase of peripheral BDNF levels with longer cocaine abstinence.

On the other hand, BDNF in prefrontal regions could have differential effects on cocaine seeking behaviors in rodents. A previous report showed that BDNF external infusions in the mPFC in rats decrease cocaine seeking behavior^71^ and improves place conditioned extinction^72^. Also, blocking BDNF effects in mPFC promotes cocaine-seeking behavior during abstinence^73^. Collectively, these findings suggest that BDNF prefrontal upregulation differently affects the cocaine-seeking behavior compared to the reward system.

Animal models show that BDNF increases due to abstinence could have a brain region-dependent effect suggesting a complex adaptive period after cocaine intake interruption. This adjustment period influenced by BDNF increases could favor craving and, according to the addictive cycle^74^, heighten the probability of further cocaine consumptions. These findings aligned with the notion that higher BDNF levels are associated with the early relapse previously discussed^51^. BDNF has been proposed as a potential addictive phase-dependent biomarker in CUD that may fluctuate in a low–high-low BDNF levels pattern along binge/intoxication-negative affect/abstinence-craving cycle (for review see Miuli et al.^55^). The reduction in variations of BDNF levels could help to prevent relapses along the addictive cycle as previously hypothesized^55^. After a possible initial increase in BDNF levels produced during early abstinence, an extended abstinence period could show a further decrease, and if no more cocaine consumption occurs, the variations over time may be reduced. In line with our proposal, an initial U-shape inverted phenomenon could be observed in BDNF levels after a sufficiently extended cocaine abstinence period and it could be linked to the U-shape craving incubation form described in animal models^75^ and recently in individuals with CUD^76^. Future studies will aid confirming a potential association between BDNF during abstinence and craving.

Early abstinence is a period characterized by high cocaine relapse rates^77^, also it is susceptible for new consumptions after abstinence periods from 6 to12 months^78^. An exhaustive description of BDNF and other neurotrophin fluctuations after cocaine withdrawal in CUD could help to identify a series of biomarkers of CUD severity. Fluctuations of neurotrophins could constitute a valuable predictor for relapse i.e., the magnitude of change in BDNF and other neurotrophins in a certain period or as a treatment-response indicator.

### Study limitations

The present work has different limitations regarding the number of studies found for the review, heterogeneity in methods to collect samples, small samples with CUD included and mainly the duration of abstinence. Additionally, the lack of statistical significance limits the generalization of those results to population of CUD, then a conclusive description of the changes in BDNF levels along abstinence remains to be determined.

### Future directions

Our work identified concise variables that should be considered for future studies evaluating BDNF levels in cocaine abstinence. Longer abstinence periods and repeated testing are necessary for an extensive description of BDNF and other neurotrophins in CUD abstinence. The monitoring of these factors throughout long-term abstinence could elucidate, at least partially, the adaptations needed to maintain abstinence over time. Additionally, these changes may be part of a brain homeostatic mechanisms and could underlay the susceptibility of relapses during cocaine abstinence.

## Conclusion

Our results showed no significant changes in BDNF levels after abstinence, which could be explained by a marked heterogeneity in the abstinence duration time among the meta-reviewed studies. The studies reviewed have heterogeneous small samples, as like only men or only women, they include a wide range in age and years of consumption, and the severity is scarcely considered in the analysis. Additionally, only one study in women considered other than BDNF neurotrophins. Considering the results presented here it is not possible to state how the neurotrophins change after abstinence.

### Supplementary Information

Supplementary Information.

## Data Availability

The datasets generated are available from the corresponding author on reasonable request.
